# Association of Adherence to the Mediterranean Diet with All-Cause Mortality in Subjects with Heart Failure

**DOI:** 10.3390/nu14040842

**Published:** 2022-02-17

**Authors:** Chih-Yun Chang, Chia-Lin Lee, Wei-Ju Liu, Jun-Sing Wang

**Affiliations:** 1 Division of Endocrinology and Metabolism, Department of Internal Medicine, Taichung Veterans General Hospital, Taichung 40705, Taiwan; cowmilk38@gmail.com (C.-Y.C.); u502107@yahoo.com.tw (C.-L.L.); 2 Department of Medical Research, Taichung Veterans General Hospital, Taichung 40705, Taiwan; 3 Department of Medicine, School of Medicine, National Yang Ming Chiao Tung University, Taipei 11221, Taiwan; 4 College of Medicine, National Chung Hsing University, Taichung 40227, Taiwan; 5 Rong Hsing Research Center for Translational Medicine, Institute of Biomedical Science, National Chung Hsing University, Taichung 40227, Taiwan

**Keywords:** Mediterranean diet, aMED, NHANES, heart failure, mortality

## Abstract

We investigated the associations of adherence to the Mediterranean diet with all-cause and cardiovascular mortality in patients with heart failure. We analyzed the National Health and Nutrition Examination Survey (NHANES) participants from 1999 to 2010, with their vital status confirmed through to the end of 2011. The alternate Mediterranean Diet Index (aMED) was used to assess study participants’ adherence to the Mediterranean diet according to information on dietary questionnaires. We conducted weighted Cox proportional hazards regression models to determine the associations of adherence to the Mediterranean diet (aMED ≥ median vs. <median) with all-cause and cardiovascular mortality in participants with a history of heart failure. A total of 832 participants were analyzed, and the median aMED was 3. After a median follow-up of 4.7 years, 319 participants had died. aMED ≥ 3 (vs. <3) was not associated with a lower risk of all-cause (adjusted HR 0.797, 95% CI 0.599–1.059, *p* = 0.116) and cardiovascular (adjusted HR 0.911, 95% CI 0.539–1.538, *p* = 0.724) mortality. The findings were consistent across several subgroup populations. Among the components of aMED, a lower intake of red/processed meat was associated with a higher risk of mortality (adjusted HR 1.406, 95% CI 1.011–1.955, *p* = 0.043). We concluded that adherence to the Mediterranean diet was not associated with a lower risk of all-cause and cardiovascular mortality in participants with a history of heart failure. The higher risk of mortality associated with a lower intake of red/processed meat deserves further investigation.

## 1. Introduction

Despite a modest decrease in the incidence of heart failure in recent decades [1,2,3], the rate of all-cause mortality remains high [3,4,5]. In a recent large cohort study [6], the 5-year survival rate was less than 50% in patients with newly diagnosed heart failure. Among the patients who were admitted to hospital at the time of diagnosis [6], the 5-year survival rate was less than 40%. This disease has been reported to be as “malignant” as some kinds of cancers [4].

In addition to pharmacologic treatment, lifestyle modification and healthy dietary patterns (such as the Mediterranean diet) have been recommended to reduce cardiovascular risk [7]. In people with obesity, adopting the Mediterranean diet was effective in improving lipid values and glycemic control, as well as achieving weight reduction [8]. Moreover, adherence to the Mediterranean diet, assessed using the alternate Mediterranean Diet Index (aMED) [9,10], has been associated with a lower risk of all-cause mortality in the general population [11,12,13].

Malnutrition is an important issue for patients with heart failure [14,15], and there have been some reports on dietary recommendations for these patients [16,17]. Nevertheless, the effect of adopting the Mediterranean diet on risk of all-cause mortality in patients with heart failure [18] is not yet clear. We conducted this study to investigate the association of adherence to the Mediterranean diet with risk of all-cause and cardiovascular mortality in patients with heart failure.

## 2. Materials and Methods

We analyzed data from NHANES participants in this study. The NHANES was conducted by the National Center for Health Statistics to assess the health and nutritional status of the general population. Relevant dietary information was collected using 24 h dietary recall questionnaires (https://wwwn.cdc.gov/nchs/nhanes/Search/DataPage.aspx?Component=Dietary&CycleBeginYear=2005, accessed on 26 January 2022). We determined dietary concordance with the Mediterranean diet using the aMED [9,10], which has been applied to the NHANES population. Information on all-cause and cardiovascular mortality was obtained by linking to the National Death Index up to the end of 2011. This study was conducted in accordance with the Declaration of Helsinki. All of the NHANES participants provided informed consent. We had our study protocol approved by the Institutional Review Board of Taichung Veterans General Hospital, Taichung, Taiwan (approval number: CE18312A) prior to conduction of the study.

### 2.1. Study Population

**Figure 1:**
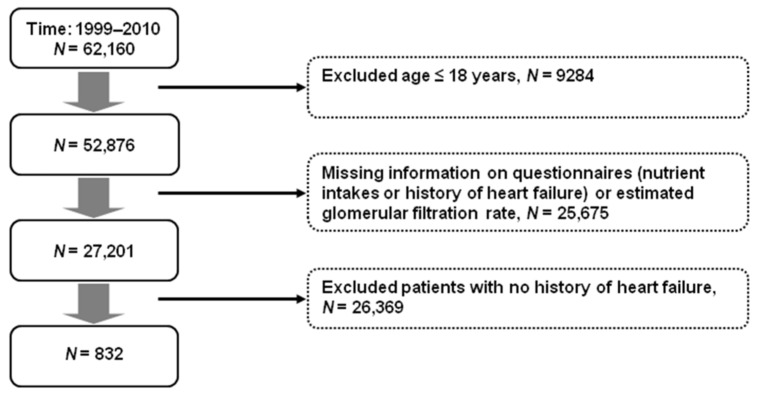
Selection process for the study population.

Figure 1 shows the selection process for the study population. From 1999 to 2010, there were 62,160 participants in the NHANES. We excluded those who were aged ≤18 years, had missing data on questionnaires for nutrient intake and renal function, or had unknown or no history of heart failure. Finally, the study population consisted of 832 participants who reported a history of heart failure.

### 2.2. Procedure and Outcome

We used aMED [9,10] to assess the study population’s dietary concordance with the Mediterranean diet. The study participants’ intakes of alcohol, red and processed meat, seafood, whole grains, legumes, nuts, fruits, vegetables (except potatoes), and ratio of monounsaturated to saturated fat were determined according to the information obtained from the 24 h dietary recall questionnaires. One point was assigned to study participants who had an intake of the aforementioned components (except for alcohol and red/processed meat) higher than the median for the population. For alcohol and red/processed meat, one point was assigned to those who had moderate alcohol consumption (10–25 g/day for men and 5–15 g/day for women) or a meat intake that was less than the median for the population. Those who did not have an intake of these components meeting the aforementioned criteria received 0 points. The maximum score was 9. The higher the aMED, the better the concordance with the Mediterranean diet. The Chronic Kidney Disease Epidemiology Collaboration equation [19] was used to determine the estimated glomerular filtration rate (eGFR). The primary outcome of this study was all-cause mortality (https://www.cdc.gov/nchs/data/nhsr/nhsr143-508.pdf, accessed on 26 January 2022). As the ankle–brachial index (ABI) has been associated with risk of mortality in patients with heart failure [20,21], the associations of aMED and ABI with all-cause and cardiovascular (including death due to heart diseases or cerebrovascular diseases) mortality were examined as well.

### 2.3. Statistical Analysis

We conducted the statistical analyses using the Statistical Analysis System survey procedures (SAS version 9.4, 2013, Cary, NC, USA) according to the analytic guidelines (https://wwwn.cdc.gov/nchs/nhanes/analyticguidelines.aspx, accessed on 26 January 2022). The study population was divided into two groups according to their aMED (≥median vs. <median) [22,23,24]. Between-group differences in categorical and continuous variables were tested using the Chi-square test and the independent sample *t*-test, respectively. We used weighted Cox proportional hazards regression models to determine the hazard ratios (HR) and 95% CI for the associations of adherence to the Mediterranean diet (aMED ≥ median vs. <median) with all-cause and cardiovascular mortality, with adjustments for age, sex, race, body mass index, smoking, systolic blood pressure, diabetes, eGFR, and daily energy intake. We conducted subgroup analyses in study participants with different body mass indexes (≥30 vs. <30 kg/m^2^), daily energy intake (≥25 vs. <25 kcal/kg/day), glucose regulation status (diabetes vs. no diabetes), and renal function (eGFR ≥ 60 vs. <60 mL/min/1.73 m^2^). The associations of the 9 components of aMED with all-cause and cardiovascular mortality were also examined. Statistical significance was confirmed with a two-sided *p* value < 0.05.

## 3. Results

### 3.1. Characteristics of the Population

Table 1 shows the baseline characteristics of the study participants according to aMED (≥3 vs. <3). Participants with an aMED ≥ 3 were older, and had a lower body mass index, a lower rate of smoking, and a better lipid profile (lower total cholesterol and triglyceride, and higher high-density lipoprotein cholesterol) compared to those who had an aMED < 3. There were also significant between-group differences in systolic and diastolic blood pressure, fasting plasma glucose, glycated hemoglobin (HbA1c), eGFR, and daily calorie intake. However, the differences were only modest.

### 3.2. Outcome

#### 3.2.1. Association with aMED (≥3 vs. <3) and ABI (≤0.9 vs. >0.9)

After a median follow-up of 4.7 years, a total of 319 participants had died (69.5 per 1000 person-years). Table 2 shows the associations of aMED (≥3 vs. <3) and ABI (≤0.9 vs. >0.9) with all-cause and cardiovascular mortality. Overall, a higher aMED was associated with a non-significantly lower risk of all-cause (adjusted HR 0.797, 95% CI 0.599–1.059, *p* = 0.116) and cardiovascular (adjusted HR 0.911, 95% CI 0.539–1.538, *p* = 0.724) mortality. In contrast, an ABI ≤ 0.9 (vs. >0.9) was associated with a significantly higher risk of all-cause (adjusted HR 2.206, 95% CI 1.412–3.447, *p* < 0.001) and cardiovascular (adjusted HR 2.027, 95% CI 1.008–4.075, *p* = 0.048) mortality. The associations of aMED (≥3 vs. <3) with all-cause and cardiovascular mortality were similar across subgroups of age, sex, body mass index, calorie intake, diabetes, and renal function (eGFR) (Table 3).

#### 3.2.2. Association with Individual Components of aMED

Table 4 shows the associations of aMED components with all-cause and cardiovascular mortality. There were no significant associations between the components of aMED with the risk of mortality, except for alcohol and red/processed meat. Moderate alcohol consumption (score = 1) was associated with a modest reduction in all-cause (HR 0.522, 95% CI 0.243–1.123, *p* = 0.095) and cardiovascular (HR 0.324, 95% CI 0.132–0.794, *p* = 0.014) mortality. In contrast, a lower intake of red/processed meat (score = 1) was associated with a significantly higher risk of all-cause mortality (HR 1.406, 95% CI 1.011–1.955, *p* = 0.043).

#### 3.2.3. Association with aMED Score for Red/Processed Meat (Lower Intake vs. Higher Intake) in Subgroups

The higher risk of all-cause mortality associated with a lower intake of red/processed meat (score = 1) was examined in subgroups. The association was noted in subgroups of age ≥65 years, female, body mass index ≥30 kg/m^2^, diabetes, and eGFR <60 mL/min/1.73 m^2^ (Table 5).

## 4. Discussion

In this study using data from the NHANES, we investigated the association of adherence to the Mediterranean diet with all-cause and cardiovascular mortality in participants with a history of heart failure. We found that aMED ≥ 3 (vs. <3) was not associated with a lower risk of all-cause (adjusted HR 0.797, 95% CI 0.599–1.059, *p* = 0.116) and cardiovascular (adjusted HR 0.911, 95% CI 0.539–1.538, *p* = 0.724) mortality (Table 2), and the findings were consistent across various subgroups (Table 3). In line with previous reports [20,21,25], a low ABI (≤0.9 vs. >0.9) was associated with a significantly higher risk of all-cause (HR 2.206, 95% CI 1.412–3.447, *p* < 0.001) and cardiovascular (HR 2.027, 95% CI 1.008–4.075, *p* = 0.048) mortality. Regarding the individual components of aMED, a lower intake of red/processed meat was associated with a higher risk of all-cause mortality (adjusted HR 1.406, 95% CI 1.011–1.955, *p* = 0.043, Table 4). Our findings showed that a dietary pattern concordance with the Mediterranean diet might not be associated with a lower risk of all-cause mortality in patients with heart failure. 

A dietary pattern concordant with the Mediterranean diet in the general population has been associated with a lower risk of incident cardiovascular diseases [26,27,28] and all-cause mortality [11,12,13]. In a well-conducted meta-analysis [29], a Mediterranean dietary pattern was associated with lower risks of cardiovascular disease incidence and mortality. Nevertheless, the associations with incident heart failure were inconsistent [26,30,31,32]. Furthermore, the effects of adherence to the Mediterranean diet on secondary prevention [33] and all-cause mortality risk [18,34] are not yet clear in patients with heart failure. In a recent study conducted in 991 patients who had an episode of acute heart failure [35], adherence to the Mediterranean diet was not associated with a significantly lower risk of all-cause mortality (HR 0.94, 95% CI 0.80–1.13, *p* = 0.50) during a mean follow-up period of 2.1 years. Our findings are consistent with previous studies [18,34,35], while these results could not support the recommendation of a Mediterranean dietary pattern to reduce risk of all-cause mortality in patients with heart failure. 

We examined the associations of individual components of the aMED with all-cause and cardiovascular mortality (Table 4). Moderate alcohol consumption (score = 1) was associated with a modest reduction in all-cause (HR 0.522, 95% CI 0.243–1.123, *p* = 0.095) and cardiovascular (HR 0.324, 95% CI 0.132–0.794, *p* = 0.014) mortality. The findings are consistent with previous studies [36,37,38]. Surprisingly, a lower intake of red/processed meat (score 1 vs. 0) was associated with a higher risk of all-cause mortality (HR 1.406, 95% CI 1.011–1.955, *p* = 0.043). Red/processed meat consumption had been associated with incident cardiovascular diseases and all-cause mortality in the general population [39,40,41], but the association was not significant in the aforementioned meta-analysis [29]. Intake of processed meat (other than red meat) had also been associated with risk of heart failure [42]. However, the association between red/processed meat intake and all-cause mortality is not yet clear in patients with heart failure. The effects of processed and unprocessed red meat on risk of mortality might be different [43,44]. Moreover, patients with heart failure might be predisposed to malnutrition [14,15], and nutritional therapy has become an important issue in treating these patients. Daily protein intake of more than 1.1 g/kg is recommended for patients with heart failure to prevent catabolism [16,17]. In a recent randomized controlled study conducted in 76 obese patients with heart failure and diabetes [45], an energy-restricted high-protein diet (30% protein, 40% carbohydrate, and 30% fat) for 3 months resulted in better control of cardiometabolic risk factors than an energy-restricted standard-protein diet (15% protein, 55% carbohydrate, and 30% fat). In our study participants whose intake of red/processed meat was lower, the percentages of daily calories from protein, carbohydrate, and fat were 14.7%, 53.2%, and 32.2%, respectively. The respective rates were 17.1%, 47.5%, and 35.4% in those with a higher intake of red/processed meat. An increase in carbohydrate intake may be associated with a higher risk of mortality [46]. We speculate that limited red meat intake in patients with heart failure might lead to inadequate protein intake, and possibly an increase in carbohydrate consumption, both of which could be associated with unfavorable outcomes in susceptible populations (e.g., patients with heart failure, the elderly, etc.) [47,48,49]. The trade-off between a decrease in protein and an increase in carbohydrate might have even more impacts on participants with diabetes, obesity, or chronic kidney disease [46,50], while a discrepancy between men and women regarding the outcomes cannot be excluded [51]. Whether replacement of animal protein with plant protein may improve survival [52] in patients with heart failure merits further investigation. 

### Limitations

Our study has some limitations. First, some measures related to heart failure were not available for analyses. Both left ventricular ejection fraction and serum N-terminal pro-brain natriuretic peptide have been associated with heart failure outcomes. Information about medical treatment for heart failure was lacking. These factors were not adjusted for in our analyses. Second, the dietary information was collected through questionnaires. Although the interviewers in the NHANES were trained to minimize data collection bias, there could have been some discrepancies between the participants’ dietary habits and the collected information, which may have confounded our results. Third, dietary concordance with the Mediterranean diet was low in our study population (median aMED = 3). Moreover, the number of study participants was relatively small. This might partly explain the null effect of aMED on the mortality risk in our results. Nevertheless, a higher concordance with the Mediterranean diet in a prospective study [35] did not influence long-term mortality in patients who experienced an episode of acute heart failure.

## 5. Conclusions

Adherence to the Mediterranean diet was not associated with risk of all-cause mortality in NHANES participants with a history of heart failure. The association of a higher risk of mortality with a lower intake of red/processed meat in the study population deserves further investigation.

## Figures and Tables

**Table 1:** Characteristics of study participants according to aMED.

	aMED		
Variables	<3	≥3	*p*
Number of participants	468	364	
Age, years	64.0 (62.3–65.6)	67.8 (65.8–69.8)	<0.001
Male, *n* (%)	275 (54.5)	204 (54.6)	0.969
Body mass index, kg/m^2^	31.2 (30.4–32.0)	30.7 (29.6–31.7)	<0.001
Systolic blood pressure, mm Hg	130.5 (127.9–133.1)	131.8 (129.0–134.7)	<0.001
Diastolic blood pressure, mm Hg	67.7 (65.5–70.0)	66.7 (64.6–68.8)	<0.001
Hypertension, *n* (%)	340 (67.5)	273 (71.9)	0.211
Smoking, *n* (%)	320 (68.0)	204 (55.8)	0.005
Total cholesterol, mg/dL	190.7 (184.9–196.6)	187.0 (182.1–192.0)	<0.001
HDL cholesterol, mg/dL	47.6 (45.8–49.4)	50.9 (48.7–53.1)	<0.001
Triglycerides, mg/dL	179.0 (163.9–194.0)	158.8 (146.3–171.4)	<0.001
Fasting plasma glucose, mg/dL	114.9 (108.8–121.1)	118.6 (112.1–125.0)	<0.001
HbA1c, %	6.03 (5.89–6.17)	6.17 (6.03–6.31)	<0.001
eGFR, mL/min/1.73 m^2^	71.1 (67.7–74.5)	69.7 (66.1–73.3)	<0.001
Daily calories, kcal/day	1748 (1636–1860)	1802 (1691–1913)	<0.001
% from carbohydrate	48.9 (47.9–49.8)	52.3 (51.2–53.5)	
% from fat	35.0 (34.1–36.0)	32.1 (31.1–33.2)	
% from protein	16.1 (15.5–16.8)	15.6 (14.9–16.3)	
With ankle–brachial index data, *n*	213	63	
Ankle–brachial index ≤ 0.9, *n* (%)	38 (19.5)	25 (18.2)	0.792

Data are presented as mean (95% CI) or *n* (%). aMED, alternative Mediterranean Diet Index. eGFR, estimated glomerular filtration rate. HbA1c, glycated hemoglobin. HDL, high-density lipoprotein.

**Table 2:** Associations of aMED and ankle–brachial index with all-cause and cardiovascular mortality.

	Adjusted HR (95% CI) ^†^	*p*
All-cause mortlity		
aMED (≥3 vs. <3)	0.797 (0.599–1.059)	0.116
Ankle–brachial index (≤0.9 vs. >0.9)	2.206 (1.412–3.447)	<0.001
Cardiovascular mortality mortality		
aMED (≥3 vs. <3)	0.911 (0.539–1.538)	0.724
Ankle–brachial index (≤0.9 vs. >0.9)	2.027 (1.008–4.075)	0.048

aMED, alternative Mediterranean Diet Index. ^†^ Adjusted for age, sex, race, body mass index, smoking, systolic blood pressure, diabetes, estimated glomerular filtration rate, and daily energy intake.

**Table 3:** Association of aMED (≥3 vs. <3) with all-cause and cardiovascular mortality in subgroups.

	All-Cause Mortality		Cardiovascular Mortality	
	Adjusted HR (95% CI) ^†^	*p*	Adjusted HR (95% CI) ^†^	*p*
Age < 65 years	0.682 (0.239–1.943)	0.470	0.529 (0.101–2.765)	0.447
Age ≥ 65 years	0.983 (0.733–1.319)	0.907	1.121 (0.651–1.929)	0.677
Male	0.720 (0.480–1.081)	0.112	1.035 (0.508–2.107)	0.924
Female	0.899 (0.592–1.366)	0.614	0.794 (0.432–1.458)	0.452
Body mass index < 30 kg/m^2^	0.740 (0.523–1.047)	0.088	0.684 (0.385–1.217)	0.194
Body mass index ≥ 30 kg/m^2^	0.846 (0.502–1.426)	0.527	1.379 (0.621–3.061)	0.425
Calorie intake < 25 kcal/kg/day	0.784 (0.556–1.106)	0.163	0.886 (0.481–1.633)	0.695
Calorie intake ≥ 25 kcal/kg/day	0.909 (0.552–1.496)	0.704	1.176 (0.506–2.734)	0.703
No diabetes	0.910 (0.663–1.249)	0.557	0.733 (0.450–1.194)	0.209
Diabetes	0.745 (0.418–1.029)	0.314	1.268 (0.494–3.253)	0.618
eGFR ≥ 60 mL/min/1.73 m^2^	0.685 (0.455–1.029)	0.068	0.935 (0.492–1.778)	0.837
eGFR < 60 mL/min/1.73 m^2^	0.780 (0.524–1.159)	0.216	0.802 (0.443–1.452)	0.462

aMED, alternative Mediterranean Diet Index. eGFR, estimated glomerular filtration rate. ^†^ Adjusted for age, sex, race, body mass index, smoking, systolic blood pressure, diabetes, eGFR, and daily energy intake.

**Table 4:** Associations of individual components of the aMED with all-cause and cardiovascular mortality.

	All-Cause Mortality		Cardiovascular Mortality	
	Adjusted HR (95% CI) ^†^	*p*	Adjusted HR (95% CI) ^†^	*p*
Alcohol score = 0	1 (reference)		1 (reference)	
Alcohol score = 1	0.522 (0.243–1.123)	0.095	0.324 (0.132–0.794)	0.014
Red/processed meat score = 0	1 (reference)		1 (reference)	
Red/processed meat score = 1	1.406 (1.011–1.955)	0.043	1.125 (0.656–1.929)	0.665
Sea food score = 0	1 (reference)		1 (reference)	
Sea food score = 1	0.960 (0.621–1.484)	0.854	0.776 (0.392–1.537)	0.463
Whole grains score = 0	1 (reference)		1 (reference)	
Whole grains score = 1	0.953 (0.713–1.274)	0.743	1.054 (0.625–1.778)	0.842
Legumes score = 0	1 (reference)		1 (reference)	
Legumes score = 1	0.764 (0.572–1.020)	0.068	0.793 (0.442–1.423)	0.432
Nuts score = 0	1 (reference)		1 (reference)	
Nuts score = 1	0.940 (0.699–1.265)	0.682	1.053 (0.642–1.728)	0.836
Fruits score = 0	1 (reference)		1 (reference)	
Fruits score = 1	0.884 (0.638–1.223)	0.451	1.060 (0.626–1.794)	0.826
Vegetables score = 0	1 (reference)		1 (reference)	
Vegetables score = 1	0.966 (0.714–1.306)	0.820	1.084 (0.711–1.653)	0.706
MUFA/SFA score = 0	1 (reference)		1 (reference)	
MUFA/SFA score = 1	0.979 (0.739–1.296)	0.879	0.804 (0.512–1.264)	0.341

aMED, alternative Mediterranean Diet Index. MUFA, monounsaturated fat. SFA, saturated fat. ^†^ Adjusted for age, sex, race, body mass index, smoking, systolic blood pressure, diabetes, estimated glomerular filtration rate, and daily energy intake.

**Table 5:** Associations of aMED score for red/processed meat (1 vs. 0) with all-cause mortality in subgroups.

	Adjusted HR (95% CI) ^†^	*p*
Overall	1.406 (1.011–1.955)	0.043
Age < 65 years	0.944 (0.363–2.452)	0.905
Age ≥ 65 years	1.524 (1.092–2.127)	0.014
Male	1.207 (0.712–2.046)	0.481
Female	1.665 (1.106–2.508)	0.015
Body mass index < 30 kg/m^2^	1.111 (0.796–1.551)	0.530
Body mass index ≥ 30 kg/m^2^	1.795 (1.001–3.219)	0.049
Calorie intake < 25 kcal/kg/day	1.473 (0.980–2.215)	0.063
Calorie intake ≥ 25 kcal/kg/day	1.559 (0.849–2.860)	0.150
No diabetes	0.979 (0.684–1.403)	0.909
Diabetes	2.318 (1.404–3.828)	0.001
eGFR ≥ 60 mL/min/1.73 m^2^	0.949 (0.542–1.660)	0.852
eGFR < 60 mL/min/1.73 m^2^	1.903 (1.298–2.791)	0.001

aMED, alternative Mediterranean Diet Index. eGFR, estimated glomerular filtration rate. Score 1 = intake of red/processed meat less than the median of the study population. ^†^ Adjusted for age, sex, race, body mass index, smoking, systolic blood pressure, diabetes, eGFR, and daily energy intake.

## Data Availability

The datasets generated and/or analyzed during the current study are available in the National Center for Health Statistics, Centers for Disease Control and Prevention (https://wwwn.cdc.gov/nchs/nhanes/Search/DataPage.aspx?Component=Dietary&CycleBeginYear=2005, accessed on 26 January 2022).
